# Multifunctional Composite Coatings Based on Photoactive Metal-Oxide Nanopowders (MgO/TiO_2_) in Hydrophobic Polymer Matrix for Stone Heritage Conservation

**DOI:** 10.3390/nano11102586

**Published:** 2021-09-30

**Authors:** Victor Fruth, Ligia Todan, Cosmin Iulian Codrea, Iuliana Poenaru, Simona Petrescu, Ludmila Aricov, Madalina Ciobanu, Luiza Jecu, Rodica Mariana Ion, Luminita Predoana

**Affiliations:** 1 ”Ilie Murgulescu” Institute of Physical Chemistry, Romanian Academy, 202 Splaiul Independentei, 060021 Bucharest, Romania; vfruth@gmail.com (V.F.); l_todan@yahoo.co.uk (L.T.); iuliana.poenaru@gmail.com (I.P.); simon_pet@yahoo.com (S.P.); aricov_ludmila@yahoo.com (L.A.); madalinabesnea@yahoo.com (M.C.); 2 Department of Science and Engineering of Oxide Materials and Nanomaterials, Faculty of Applied Chemistry and Materials Science, University Politehnica of Bucharest, 060042 Bucharest, Romania; 3 National Institute for Research & Development in Chemistry and Petrochemistry INCDCP-ICECHIM, 202 Splaiul Independentei, 060021 Bucharest, Romania; luizajecu@yahoo.com (L.J.); rodica_ion2000@yahoo.co.uk (R.M.I.)

**Keywords:** stone heritage, multifunctional composite, oxide nanopowders, sodium polyacrylate, sol–gel, layer-by-layer

## Abstract

Multifunctional composite coatings composed of metal oxide nanoparticles dispersed in polymer matrices are an advanced solution to solve the problem of stone heritage deterioration. Their innovative design is meant to be stable, durable, transparent, easy to apply and remove, non-toxic, hydrophobic, and permeable. Coating formulations for the protection of buildings and monuments have been intensively researched lately. Such formulations are based on multifunctional composite coatings incorporating metal oxides. The present work aims to combine the hydrophobic properties of sodium polyacrylate (NaPAC_16_) with the antimicrobial effectiveness, with promising antimicrobial results even in the absence of light, and good compatibility of MgO (a safe to use, low cost and environmentally friendly material) and TiO_2_ (with antibacterial and antifungal properties), in order to develop coatings for stone materials protection. MgO (pure phase periclase) and TiO_2_ (pure phase anatase) nanopowders were prepared through sol–gel method, specifically routes. Aqueous dispersions of hydrophobically modified polymer (NaPAC_16_, polyacrylic acid sodium salt) and MgO/TiO_2_ nanopowders were deposited through layer-by-layer dip coating technique on glass slides and through immersion on stone fragments closely resembling the mosaic stone from the fourth century AD Roman Mosaic Edifice, from Constanta, Romania. The oxide nanopowders were characterized by: Thermal analysis (TG/DTA), scanning electron microscopy (SEM), X-ray diffraction (XRD), BET specific surface area and porosity, and UV–Vis spectroscopy for band gap determination. An aqueous dispersion of modified polyacrylate polymer and oxide nanopowders was deposited on different substrates (glass slides, red bricks, gypsum mortars). Film hydrophobicity was verified by contact angle measurements. The colour parameters were evaluated. Photocatalytic and antimicrobial activity of the powders and composite coatings were tested.

## 1. Introduction

Conservation of the patrimony, a legacy to our future, is of high priority because of their incalculable symbolic value and economical potential. A variety of factors, such as stone specific properties, conservation condition, degradation mechanism, and environmental factors, should be taken into account to select the adequate materials and procedures for a suitable conservation treatment [1]. Stone heritage buildings are prone to weathering caused by microorganisms and environmental pollutants attack. The application of self-cleaning protective treatments on historically and architecturally significant stone surfaces can improve the conservation and maintenance of this cultural patrimony [2]. Cultural heritage conservation was previously based on the traditional conservation and restoration treatment methods, such as the use of synthetic polymers, which do not have durable performance and are not compatible with the substrate [1]. 

Lately hybrid and composite materials with innovative surface structure were designed and produced for the protection of stone heritage monuments offering enhanced proprieties [3]. Innovative applications have been designed by using nanoparticles, such as in building materials achieving environmental pollution remediation, self-cleaning, and antimicrobial activity. In order to be used in this type of applications, nanoparticles must possess the following characteristics: stability, photoactivity, chemical inactivity, nontoxicity, suitability towards visible or near UV light, water repellence, low cost, etc. Nanomaterials such as SiO_2_ nanoparticles, CaCO_3_, and clay are some of the most frequent materials used in the consolidation of heritage archaeological monuments. Ag, ZnO, and TiO_2_ nanoparticles are the most frequent nanomaterials in surface protection and preservation [2]. 

In stone heritage conservation applications, surface treatments must fulfil specific requirements. In their design, one must consider that stone colour should not be altered in order to preserve the aesthetical aspect and the coating treatment should be reversible. The natural pores of the stone should not be plugged and the coating itself should be permeable to water vapor in order to avoid moisture and soluble salts accumulation, and subsequent shear stresses, at the interface between the treated zone and the untreated stone beneath [4]. 

Photo-catalytic and antimicrobial oxide nanoparticles, such as the ones mentioned above [2], embedded in different coating systems were prepared with the aim of preparing a multi-functional film with self-cleaning and anti-bacterial properties [1,5,6].

In this context, coatings obtained from magnesium oxide incorporated in an alkyd resin (with negligible anti-bacterial properties) displayed very good catalytic properties in degrading methyl violet [7] and bacterial inhibition [5]. Numerous techniques are used for the preparation of MgO powders, such as Mg(OH)_2_ dehydration, decomposition of different magnesium precursors using thermal evaporation, sol–gel, hydrothermal, flame spray pyrolysis and surfactant methods [8,9,10,11,12]. Sol–gel method can produce MgO with large size distribution and minimum constraints [13]. In case of wide band gap materials, the electronic, optical, and chemical properties depend on the defects in their crystal structure, such as point defects and step edges [14,15]. Undertaken studies have evidenced that in MgO nanocrystalline powders more such defects can be generated than in macrocrystalline powder. There is a difference in the luminescent properties of MgO nanopowders and that of macropowder or MgO single crystal. Significant blue shift was observed in nanopowder MgO samples [16]. Optical properties and optical degradation of MgO in all crystalline forms (crystal, macro and nanoparticles) in the spectral region from 250 to 700 nm is determined by point defects in the oxygen sublattice (so-called F-type centers) [17,18]. 

In our study, MgO and TiO_2_, the former being used as a reference oxide, were chosen for the formulation of non-toxic, multifunctional coatings with self-cleaning and antimicrobial property and were synthesized by sol–gel method, which have the potential for obtaining a uniform distribution of the inorganic nano-dispersed phase in the organic-inorganic composites, even at the molecular level. Thus, the sol–gel method allows the regulation of the structure by controlling the conditions of hydrolysis-condensation reactions [19]. Furthermore, the sol–gel method is a promising option for the synthesis of nanostructured materials due to several reasons, such as low reaction temperature, simple inexpensive process, producing nano-size oxides with large surface area, narrow size distribution, and generating crystal defects [20].

Known for its highly efficient photocatalytic activity, nano-TiO_2_ has been intensely researched as an active component integrated in protective coatings [21,22,23,24,25].

Nevertheless, dispersion and agglomeration of TiO_2_ nano-particles in aqueous solutions is especially problematic due to the agglomeration or the segregation of TiO_2_ particles, which are often the result with conventional methods for fabricating titania–polymer nanocomposites [26].

The term ex-situ refers to mixing of the polymer matrix with the inorganic particles either in solution or by mechanical means, whereas the term in-situ is used when the particles are synthesized within the polymer matrix [27].

Layer-by-layer deposition technique represents one of the most recent methods for obtaining polyelectrolyte thin films of nanometric and lesser dimensions. For this reason, this technique and its products can be included in the top field of nanotechnology. It was shown that the deposition of layers on different substrates can be performed using solutions of polymers with opposite electric charge, ensuring, thus, a control at molecular level of the resulted multilayer structure [28].

This technique has been used to coat different substrates. In this way, organic-inorganic hybrid films were prepared including ZrO_2_ nanoparticles that could control the surface roughness and hydrophobicity [29].

Advanced composite materials with special surface structures were produced for stone protection, developing enhanced hydrophobicity and even superhydrophobicity [30,31].

Water soluble hydrophobically modified sodium polyacrylates (NaPACns) are amphiphilic macromolecular compounds having a hydrophilic backbone on which hydrophobic groups are chemical grafted. This leads to unique materials that are useful in a variety of applications. The special properties of NaPACns are given by the interplay of electrostatic (repulsion and attraction) and hydrophobic interactions [32,33]. The use and investigation of these materials is justified by the different fields in which they can be applied. In early studies reported by our group, it was first demonstrated that NaPACns have hosting properties for hydrophobic compounds [34,35], anticorrosive properties [36], water-repellent properties [37], long-term water-repellent properties [38], and antimicrobial properties [39]. Due to these characteristics, this multifunctional polymer was chosen to be combined, for the first time, with oxide nanoparticles to develop coatings for stone materials protection.

The hydrophobically modified sodium polyacrylate (NaPAC_16_) forms intramolecular aggregates [32] which ensures optimal suspension stability and fluidity, thus facilitating the film deposition/coating application. Additionally, it was shown that NaPAC_16_ films’ hydrophobicity is enhanced in time through natural aging due to water release from their structure [38], as described also in the subsequent patent application [40]. 

MgO has antimicrobial activity without photo-activation [41] and is able to deactivate both Gram-negative and Gram-positive bacteria through the leakage of intracellular contents and, eventually, bacterial death in the presence of MgO nanoparticles [42,43]. Their antibacterial effect is particle size dependent and dosage dependent and increases with lower particle size and higher MgO concentration [41].

Nano-TiO_2_ is known for becoming very reactive under UV radiation and having a strong antibacterial activity due to the reactive oxygen species (ROS) it produces [44], with strong oxidizing property resulting in a damaged cell wall and membranes [45], but some studies have also indicated toxicity towards bacteria under sunlight [44], to both Gram bacterial groups [45]. The reactivity of nanoparticles is determined by their specific surface area, which can be lowered by aggregation, thus making anatase more reactive than rutile, which has a higher tendency to aggregate [44].

Nano-TiO_2_ can inactivate bacteria, when in an acidic medium, due to the attraction generated by the opposite electrostatic charges of bacterial cells and TiO_2_ nanoparticles, disturbing the colony-forming ability of bacteria [45].

The main mechanisms proposed for the antimicrobial effect are, namely, the formation of ROS (hydrogen peroxide, superoxide radical, and hydroxyl radical), the interaction of nanoparticles with microorganisms, subsequently leading to damaged bacterial cell, and an alkaline effect [41]. When exposed to ultraviolet (UV), TiO_2_ can produce active oxygen species, depending on its crystal structure and UV light intensity. TiO_2_ antimicrobial activity is related to the capacity of these ROS to disrupt the outer membrane of bacteria, more specifically, the phospholipids, proteins and lipopolysaccharides presented there and inactivating, thus, the bacteria [46].

Nanoparticles smaller than 100 nm exhibit significantly enhanced antimicrobial activities due to the increased surface area which determines the number of reactive groups on the particle surface [47].

The present research aims to combine the hydrophobic properties of sodium polyacrylate (NaPAC_16_) with the antimicrobial effectiveness, with promising antimicrobial results even in the absence of light, and good compatibility of MgO (safe to use, low cost and environmentally friendly material) and TiO_2_ (also with antimicrobial properties), in order to develop coatings for stone materials protection [40]. 

## 2. Materials and Methods

### 2.1. Oxide Preparation and Characterization

The oxide powders were synthesized by sol–gel method. This method is a versatile method because it presents advantages, such as the processing temperature is relatively low, the obtained nanomaterials have high purity, offers the possibility of controlling stoichiometry, and obtaining nanocomposite materials, materials of different shapes, and/or with predetermined structure [48]. The MgO nano-powder were synthesized through the sol–gel route starting from magnesium nitride hexahydrate (Mg(NO_3_)_2_^.^6H_2_O) precursor and using ethyl alcohol (C_2_H_5_OH) as solvent, H_2_O as hydrolysis reagent and ammonia as catalyst. The detailed synthesis conditions can be found in Todan et al. [49]. 

For TiO_2_ nano-powder synthesis, titanium tetraisopropoxide (Merck, Hohenbrunn, Germany) [TIP = Ti(O-i-C_3_H_7_)_4_] was used as TiO_2_ precursor, the parental alcohol was chosen as solvent, and water for hydrolysis. To get a pH of 10, NH_4_OH was added into the solution. The compositions in molar ratio were Ti alkoxide:ROH:H_2_O:catalyst (NH_4_OH) = 1:36.5:1:0.003. 

For both prepared oxides the starting solutions were homogenized under vigorous stirring at room temperature for 1 h. The resulting oxide powders were separated from solution through filtration, washed with distilled water, dried, and then, according to thermal analysis results, thermally treated at 450 °C, 1 h plateau, with a heating rate of 1 °C/min. 

Phase constitution of the synthesized nano-powders was determined by X-ray diffraction (XRD) scans using Ultima IV X-ray Diffractometer (Rigaku, Tokyo, Japan), Cu Kα radiation. Nano-powder particle morphology was investigated by scanning electron microscopy (SEM) imaging using the FEI Quanta 3D FEG model, operating at 20 kV in high vacuum mode, while the powder surface physical properties (BET surface area and porosity) were determined from Brunauer–Emmett–Teller (BET) measurements through nitrogen adsorption analysis at −196 °C using a Micromeritics ASAP 2020 analyzer.

The ultraviolet–visible (UV–Vis) spectra were recorded on a JASCO V570 spectrophotometer, using spectralon as reference. Measurements were carried out in the range 800–200 nm. The result of the optical absorption energies measurements for MgO samples were obtained using the Kubelka-Munk function by plotting (F(R)hν)^2^ versus photon energy (eV).

Antimicrobial activity of MgO and TiO_2_ nanopowders was assessed through measurement of the inhibition of microbial growth in a suspension of microorganisms in broth culture media, by measuring the absorbance at 24 h, at 600 nm, with the help of the plate reader, Clariostar.

The bacterial strain *Staphylococcus aureus (S. aureus)* was transplanted onto the TSA medium and incubated for 24 h at 37 °C. The inoculum used was a suspension in sterile physiological water made from a fresh culture of 18–24 h (4–5 isolated colonies), developed on solid TSA medium. The inoculum density of 1–3 × 105 CFU/mL was adjusted spectrophotometrically by measuring the absorbance at 600 nm. The tests were performed in 100 mL Erlenmeyer flasks with 30 mL Mueller-Hinton broth medium, which were inoculated with 0.6 mL bacterial suspension. The nanoparticle samples were introduced into the culture medium inoculated with *S. aureus* and subsequently incubated for 24 h at 37 °C and 125 rpm. The control was prepared in the same way, but without the introduction of the nanoparticle sample. After 24 h of incubation, 200 µL was taken from each sample and distributed in the 96-well plate. Sampling was performed 5 times for each sample. The biological control does not contain nanoparticle powders. Subsequently, measurement of absorbance with the Clariostar plate reader was performed.

The following culture media from Scharlau were used: Tryptic Soy Agar—TSA (15 g/L casein; 5 g/L peptone; 5 g/L NaCl; 15 g/L agar). Muller Hinton broth (17.5 g/L peptone; 1.5 g/L starch; 2 g/L meat extract). Both with pH 7.3 ± 0.1 at 25 °C.

### 2.2. Composite Coatings Preparation and Characterization

Polyacrylic acid solution (abt. 25%) from Wako, and hexadecylamine (98%), dicyclohexylcarbodiimide, anhydrous N-methyl-2-pyrrolidinone, sodium hydroxide, methanol, poly (ethylenimine) aqueous solution (PEI, 50 wt.%, Mw of 75,000), and poly (diallyldimethylammonium chloride) aqueous solution (PDADMAC, 23 wt.%, Mw 100,000–200,000) were supplied by Merck KGaA, Darmstadt, Germany.

The hydrophobically modified sodium polyacrylate (NaPAC_16_) was synthesized by functionalization of polyacrylic acid (PAA) with hexadecylamine, as reported elsewhere [32]. The NaPAC_16_ was used as dispersion media for the oxide nanoparticles.

Hydrophobic polymer-oxide composite coatings were prepared from suspensions of oxide powders (0.5 wt.%) in NaPAC_16_ aqueous solution (0.1 wt.%).

Dipping Robot DR-3, Riegler and Kirstein GmbH was used to create layer-by-layer (LbL) polymer-oxide coatings. The LbL procedure on glass substrate was reported in a previous study [37]. The glass support was functionalized with poly (ethylenimine)—PEI—in order to obtain a cationic end-layer. Then, on the functionalized glass, five layers of thin films were obtained using electrostatic deposition of NaPAC_16_ or (NaPAC_16_ + oxide) and poly (diallylmethylammonium chloride)—PDADMAC. The number of layers was chosen to achieve a hydrophobic surface [37].

The wettability of glass, stone, and deposited film surfaces was tested by static contact angle measurements using an Easy Drop Shape Analyzer (DSA1, KRÜSS GmbH). The measurements were performed at room temperature in air, via sessile drop method. The samples were positioned on a plane stage and Millipore water was dripped using a stainless-steel needle with an outer diameter of 0.5 mm (drop volume of 3 µL). All contact angle values are an average of three measurements. 

For topographic analyses, a Profilm3D (Filmetrics, San Diego, CA, USA) optical profilometer with White Light Interferometry (WLI) was used. The analyses were performed using ×10 lens and a camera of 2592 × 1944 (5 megapixels). Performance specifications in measuring surface profiles include step height accuracy of 0.7% and thickness range of 50 nm to 10 mm. Images have been processed with Profilm Software (Filmetrics, San Diego, CA, USA). 

The chromatic parameters for the treated bricks were recorded with a Konica Minolta CR-410 colorimeter. In order to determine the chromatic parameters, three measurements were performed both for the reference and for the treated samples and their average was made. The colour parameters are: L*—degree of colour lightness, a*—green-red chromatic coordinates, and b*—blue-yellow chromatic coordinates. The determined parameters are: ΔL (difference in brightness), Δa (chromatic deviation of coordinates a, red and green), Δb (chromatic deviation of coordinates b, yellow and blue), and ΔE (colour variation and stability).

ΔE = [(ΔL*)^2^ + (Δa*)^2^ + (Δb*)^2^]^1/2^ (1)

where ΔL*, Δa*, and Δb* are the differences between the sample specimens and the reference specimens [50]. 

Antimicrobial activity of polymer-oxide suspensions was assessed by the diameter of growth inhibition of both bacterial strains (*Staphylococcus aureus*) and fungal strains (*Candida albicans, Aspergillus niger*), on Mueller-Hinton agar medium (for both bacterial and fungi strains), in light and dark conditions through disk diffusion qualitative method. Disk diffusion testing method in which paper disks, each saturated with the substance of interest, are positioned on the surface of agar medium previously inoculated with a bacterial isolate. 

Test microorganisms (*Staphylococcus aureus, Candida albicans, Aspergillus niger*) were streaked across a Mueller-Hinton agar plate using aseptic technique, with a sterile swab to form an adequately uniform bacterial lawn, using an inoculum with a bacterial suspension density of 1–3 × 108 CFU/mL and a fungal suspension of 1–5 × 106 CFU/mL (both at a density of 0.5 McFarland Standard). Subsequently, 10 µL of each compound, kept in the ultrasonic bath for 1 h before testing, was added as a spot. Additionally, sterile paper discs were immersed in the sample, kept for 1 h for soaking, and then placed with sterile tweezers on the bacterial culture on agar medium. Petri dishes containing inoculated Mueller-Hinton agar were incubated for 18–24 h at 37 °C for bacterial strains, and 72 h at 28 °C for fungal strains. The samples were analyzed in duplicates. Reading of growth inhibition results was performed by measuring, with the help of a graduated ruler, the radius of microbial growth inhibition area on two perpendicular axes. The following culture media from Scharlau was used: Tryptic Soy Agar—TSA (15 g/L casein; 5 g/L peptone; 5 g/L NaCl; 15 g/L agar. Muller Hinton broth (17.5 g/L peptone; 1.5 g/L starch; 2 g/L meat extract), Muller Hinton agar (17.5 g/L peptone; 1.5 g/L starch; 2 g/L meat extract, 17 g/L agar). Both with pH 7.3 ± 0.1 at 25 °C.

## 3. Results

### 3.1. MgO and TiO_2_ Nano-Powders

MgO and TiO_2_ nano-powders were obtained by the sol–gel method, as presented above in part 2. Materials and methods. From synthesis, white powder for MgO and a light-yellow powder for TiO_2_ were obtained.

According to thermal analysis results (TG/DTA, not shown here), those powders were thermally treated at 450 °C, with a heating rate of 1 °C/min and 1 h plateau.

The thermally treated powders were analyzed by SEM, XRD, and BET investigations to bring information about the morphologies and structure of the studied samples.

**Figure 1:**
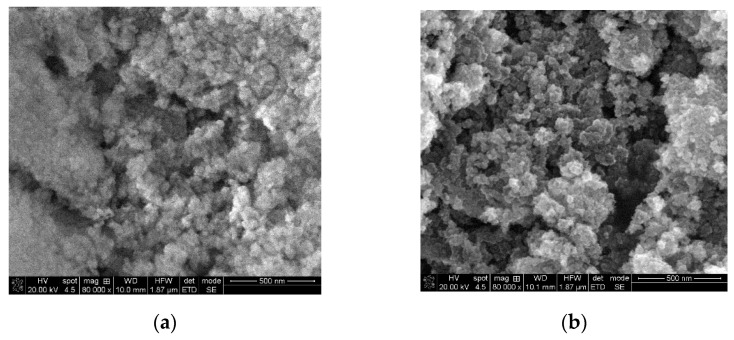
SEM images of powders: (**a**) MgO and (**b**) TiO_2_.

The SEM images of the thermally treated samples are presented in Figure 1, and they indicate that MgO powder consist of highly porous agglomerates of nearly spherical nanoparticles, TiO_2_ powder consists of agglomerated, quasi-irregular round-shaped nanoparticle, with range of dimensions between 24 to 56 nm in case of both oxides.

**Figure 2:**
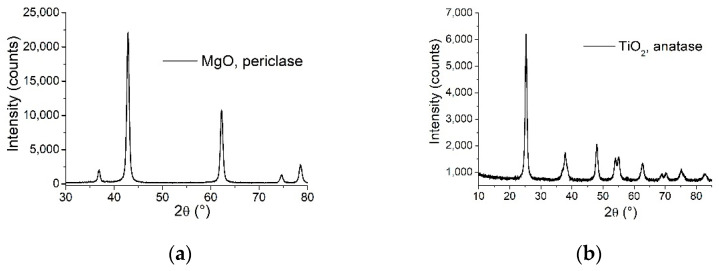
XRD patterns of powders: (**a**) MgO and (**b**) TiO_2_.

The XRD patterns of the two powder samples presented in Figure 2 indicate that both MgO and TiO_2_ samples are pure phase, consisting of periclase (JCPDS card no. 045-0946) and anatase (JCPDS card no. 21-1272) phase, respectively. The nanocrystalline cubic periclase phase was confirmed by 2θ = 36.87, 42.85, 62.25, 74.63, 78.56 corresponding to (111), (200), (220), (311), (222) reflections, in concordance with the reported data above, the lattice parameters are a = b = c = 4.2172 Ǻ, α = β = γ = 90°, and the crystallite size 158.7 Ǻ according to Williamson-Hall method. The nanocrystalline anatase phase was confirmed by 2θ = 25.25, 37.8, 47.96, 53.93, 54.99, 62.86, 68.66, 70.25, 75.16, 82.78, corresponding to (101), (004), (200), (105), (211), (204), (116), (220), (215), (224) reflections and the lattice parameters are a = b = 3.7889 Ǻ, c = 9.4984 Ǻ, α = β = γ = 90°, and the crystallite size 104 Ǻ according to Williamson-Hall method.

The BET surface area analysis results are presented in Table 1. Although the samples have comparable surface area, MgO nano-powders are significantly more porous.

It is well known that MgO in bulk phase has a band gap of about 7.8 eV [16], which suggests an insulator-like behavior [51]. Diffuse reflectance UV–Vis spectra were recorded for the synthesized MgO powders, the reflectance measurements were converted to absorption spectra using the Kubelka-Munk function in order to calculate the band gap energy using Tauc method for direct band gap semiconductors. The nanostructured particles obtained by sol–gel method decreased the band gap to 4.7 eV (see Table 2), conferring its photocatalytic properties in methyl orange (MO) photodegradation. For TiO_2_, the band gap determined similarly was 3.15 eV. The estimated band gaps of the oxide powders indicate the fact that they present activity in the UV domain, explaining, thus, the high photocatalytic efficiency under UV irradiation. The photocatalytic activity of the materials was evaluated by the spectrophotometer monitoring the MO degradation in aqueous solution, in the presence of oxide powders, after irradiation with UV/visible light. 10 mL MO solution 1 × 10^−5^ M was introduced in quartz glass vessels. The powders were added, and it was kept under magnetic stirring in the dark for 30 min in order to stabilize the adsorption of MO dye over the photocatalyst surface. Then, the reactor was exposed to irradiation and aliquots from the solution were withdrawn at specific time intervals. The concentration of the MO remaining after illumination in the solution was determined using a spectrophotometer method at 464 nm absorption maximum, characteristic for MO molecule. The photodegradation performance of the process was expressed in terms of decolorization efficiency in the presence of MgO and TiO_2_ powders, estimated by the decrease of the 464 nm band in time. The photocatalytic activity is higher in the UV domain in accordance with UV–Vis absorption spectra of the powders. The results are presented in Table 2 and one can see that after three hours over 80% of the dye is degraded by both oxides, TiO_2_ being more efficient. The high photocatalytic activity of the materials can be attributed to their raised absorption capacity provided by a high surface area (see Table 1), a process that ensures the presence of the MO molecule on the surface, in the immediate vicinity of the active centers, thus facilitating the photodegradation process. TiO_2_ has a higher surface area and, as mentioned in XRD analysis, is in anatase form, these resulting in the higher photocatalytic activity compared to MgO.

### 3.2. Antimicrobial Activity of MgO- and TiO_2_-Based Suspensions and Polymer-Oxide Suspensions (NaPAC_16_-MgO and NaPAC_16_-TiO_2_)

To evaluate the antimicrobial activity, the absorbance at 24 h, at 600 nm, was measured with the help of the plate reader, Clariostar. 

Because the nanoparticle powders also cause a disturbance of the culture medium, they were immersed separately in the culture medium, without inoculating the *S. aureus* strain. Thus, we also read their absorbance. The difference is made between the OD of the samples with *S. aureus* and the OD of the controls (without bacteria), and in the end it is compared with the biological control of S. aureus.

Results presented in Table 3 showed, in concordance with earlier scientific research, that MgO and TiO_2_ nanopowders exhibit antimicrobial activity. The MgO sample determined the highest inhibition of the bacterial strain (*S. aureus*), followed by the TiO_2_ sample. All values are an average of three measurements.

Experimental data obtained for polymer-oxide suspensions presented in Table 4 indicate also antimicrobial activity. This was assessed by the diameter of the growth inhibition zone of bacterial and fungal strains formed around the inoculation area (spot or paper disc). Results show that the investigated polymer-oxide suspensions (0.1% NaPAC_16_ and 0.5% oxide in water) have microbial activity against *Staphylococcus aureus*, *Candida albicans*, and *Aspergillus niger*. The highest growth inhibitory activity was observed in the *S. aureus* strain for both suspensions. Spot application of polymer-oxide suspensions showed larger growth inhibitory zones.

Antimicrobial activity of both nanoparticle powders and polymer-oxide suspensions were demonstrated. 

### 3.3. MgO- and TiO_2_-Based Films on Glass Substrates

**Figure 3:**
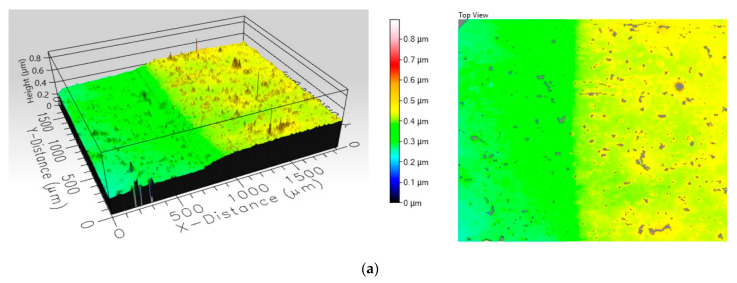

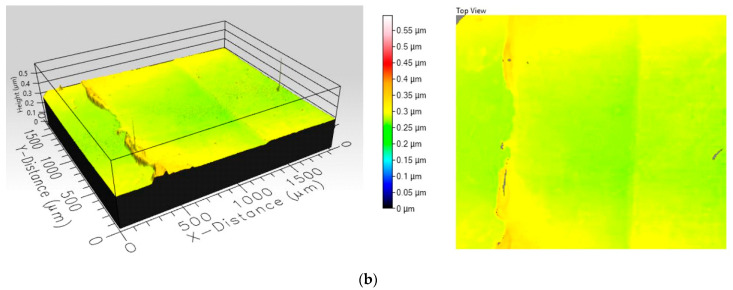
Surface profile of composite films deposited on glass slide substrates: (**a**) MgO—NaPAC_16_ and (**b**) TiO_2_—NaPAC_16_.

Images from Figure 3 show the topographic profile made on their surface by optical profilometry measurements. These composite coatings were investigated by wide area film surface topography and profiles were mapped by white light interferometry technique using a Filmetrics Profilm 3D optical profiler. The images obtained indicate a difference in roughness between the two samples, as seen in Table 5. Analyzing the 3D profiles and the colour scale, it can be seen that the film incorporating MgO nanopowders is significantly rougher than the film containing TiO_2_. The increased roughness observed in the case of the composite film incorporating MgO nanoparticles is explained by the accentuated tendency of agglomeration of MgO nanoparticles in the polymer suspension.

Area roughness of composite films was evaluated using Root mean square (RMS) height measurement, according to the ISO 25178 standard, provided by the Filmetrics software for the Filmetrics Profilm 3D optical profiler. As RMS height increase, it is expected that contact angle values increase also.

The surface hydrophobicity is measured in terms of the contact angle of water drops to the surface. Objects with hydrophobic behavior are considered self-cleansing materials. The main two concepts in the production of self-cleaning surfaces are the production of surfaces with repellent properties and the capacity of surfaces to break down or decompose dirt [52], which is the object of the photocatalysis experiments presented below. 

The static contact angle and surface hydrophobicity determined on coated and uncoated glass slide substrate are presented in Table 6. For the composite material obtained from MgO nanoparticles (0.5%) in NaPAC_16_ (0.1%) solution, the mean value of the contact angle 98.79° shows a hydrophobic behavior, while the mean contact angle of the films based on TiO_2_ situates the material to the lowest limit of the hydrophobic domain. 

The oxide nano-powder is the photoreactive component; therefore, the powder surface must be exposed in direct contact with the solution to allow the photocatalytic reaction to take place. The size of the powder aggregates and their distribution in the polymer film will therefore impact on the photodegradation efficiency of the coating. Increased film roughness is an indicator that particle aggregates stick on the polymer surface, so the photodegradation efficiency results are consistent with the surface profile observations.

The photocatalytic activity of the films containing 0.5 mg/mL oxide nanopowders deposited on glass substrate was evaluated in the same way as that of the powders, which was shown above (Table 2). In Table 7, the values obtained for methyl orange photodegradation due to the activity of composite films are presented. 

Results show that MgO-based films exhibit the best activity (49.13%) under UV light exposure after 5 h of irradiation. The resulted percentage photodegradation of MO obviously show that photocatalytic efficiency of films strongly depends on thickness, crystallite size, roughness, etc. Results denote that decreases in the film thickness, as an effect of powders calcination, lead to an increased crystallinity and crystallite size on the films and, consequently, the photocatalytic efficiency decreases. 

### 3.4. MgO- and TiO_2_-Based Films on Stone Substrates

After testing on glass slides (quasi-ideal surfaces, topographically reference), composite coatings were made by immersion for 15 min, on fragments of red bricks and natural historical stone (limestone and marble identical with that from the Triumphal Monument Adamclisi and the Roman Mosaic Building). Colorimetry and contact angle measurements were carried out to determine the changes in appearance/colour and degree of wetting of the intervened surfaces after the application of protective coatings.

Testing of composite films were conducted on natural historical limestone with identical compositions to Adamclisi stone fragments. The values of static contact angles on the limestone fragments covered with MgO-NaPAC_16_ have an average value of three measurements of 106.51°, whereas fragments covered with TiO_2_-NaPAC_16_ have an average value of 107.72°. Subsequently, the values of the static contact angles for the obtained composite films were higher than 90°, suggesting hydrophobic behavior. In Table 8, colorimetric parameters measured on these fragments before and after immersion coating are presented.

The ΔE parameter evaluates the total colour change, its variation between 0 and 0.2 indicating visually imperceptible changes. Between 0.2 and 2 there is a minor colour difference. At values higher than 2 the colour changes are visible, and over 6 the colour is severely affected or even different. The results from the point of view of colour stability, as presented in the Table 9, show that composite films slightly increase the brightness parameter (ΔL), but do not bring significant colour changes (ΔE < 2).

**Figure 4:**
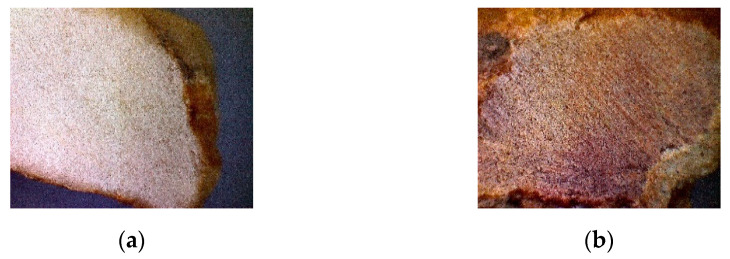
The 20× magnification images captured on polished surfaces of mosaic stone fragments: (**a**) M1(a) white marble, composition 100% CaCO_3_ and (**b**) M1(b) dolomitic marble, reddish-brown, CaMg(CO_3_)_2_ with Fe_2_O_3_ impurities).

In the case of mosaic stone fragments in the Figure 4, images show mechanically polished stone fragments, with rather compact and homogenous surfaces. 

With the testing of composite films on the mosaic stone fragments, the values of static contact angles on the fragments covered with MgO-NaPAC_16_ have an average value of three measurements of 105.03° on white fragments and 108.21° on brown fragments. In the case of fragments covered with TiO_2_-NaPAC_16_, there is an average value of 111.12° on white fragments and 100.38° on brown fragments. As in the previous cases, the values of the static contact angles for the mentioned composite films were higher than 90°, suggesting a hydrophobic behavior.

Regarding the colour stability, when reviewing the results of composite films on the mosaic stone fragments presented in Table 9, it was observed that composite films incorporating MgO nanopowders have the best transparency regardless of the nature and initial colour of the substrate until, as in precedent case, due to the uniform distribution of nanoparticles, TiO_2_-based coatings slightly increase the brightness parameter (ΔL), having a mattifying and whitening effect, but do not bring significant colour changes (ΔE < 2).

From the testing of composite films on red brick samples, static contact angles could not be measured because there was an instantaneous absorption of water droplets due to the high porosity of the substrate. 

From the point of view of colour stability, results of composite films on red bricks samples are presented in Table 10, whereby it was observed that composite films incorporating MgO nanopowders have the best transparency regardless of the nature and initial colour of the substrate, with a value of 0.18 (colour stability parameter, ΔE < 0.2). Due to the uniform distribution of nanoparticles, TiO_2_-based coatings slightly increase the brightness parameter (ΔL), especially on colored stone substrates, having a mattifying and whitening effect, with a value of 0.39, but do not bring significant colour changes (ΔE < 2).

## 4. Discussion

The self-cleaning functionality of the studied coatings is determined through the combined effect of the self-sterilization, photocatalysis, and surface hydrophobicity properties.

Tests performed on glass substrate show that the photodegradation efficiency depends on the degree of exposure of the surface of the powder particles (the difference is discernible from the profile and roughness of the films, that is, the MgO film is rougher and has significantly higher photodegradation efficiency).

A difference in composite coatings roughness was demonstrated by the surface topography profiles, using RMS height measurement.

From the point of view of colour stability, it was observed that composite films incorporating MgO nanopowders have the best transparency regardless of the nature and initial colour of the substrate (colour stability parameter, ΔE < 0.2). Due to the uniform distribution of nanoparticles, TiO_2_-based coatings slightly increase the brightness parameter (ΔL), especially on colored stone substrate, having a mattifying and whitening effect, but do not bring significant colour changes (ΔE < 2).

The contact angle tests performed on the glass slats, and on the limestone and mortar stone fragments from Adamclisi, show that the effect of the protective coatings on the wetting properties of the surfaces depends on the properties of the substrate (initial roughness and porosity). 

Antimicrobial activity of oxide nanopowder suspensions against *S. aureus* in liquid growth medium was highest for the MgO sample. For polymer-oxide suspensions tested on agar growth medium, the highest growth inhibition was observed for the NaPAC_16_-TiO_2_ sample against *S. aureus*, and for NaPAC_16_-MgO sample against *C. albicans* and *A. niger*.

## 5. Conclusions

Our research demonstrated the feasibility of using sodium polyacrylate (NaPAC_16_) and nano MgO and TiO_2_ coatings on historic stone surfaces, red brick material, and limestone samples, with a composition similar to the one of the stone from Adamclisi Tropaeum Traiani Monument and Roman Mosaic Constanta, in order to reduce the degradation process. The above mentioned nanocrystalline oxides with dimensions under 100 nm have a better photocatalytic and biological activity than macrocrystalline powders, insuring a homogeneous composition with the polymer. The most convenient way of synthesizing the nano oxides was the sol–gel route due to its low cost, ease of fabrication at low temperature, and being able to tailor their properties for specific applications. 

It was shown that the obtained films have self-cleaning properties degrading the dye under UV exposure and have effective antimicrobial activity. Results also show good water repellence, demonstrating hydrophobic properties, and the colour change had negligible variations. This research shows future perspectives in stone heritage protection through the use of innovative composite coatings. Future research will follow the behavior of these protective coatings over longer periods of time. 

## 6. Patents

National patent application with title Process for obtaining nanocomposite films intended to protect the lithic architectural components of the cultural heritage (Procedeu de obținere a unor pelicule nanocompozite destinate protejării componentelor arhitecturale litice ale patrimoniului cultural). OSIM nr. A/00350/10.06.2019 Grant of invention patent no. 134390 August 2021.

## Figures and Tables

**Table 1:** TiO_2_ and MgO powders surface properties determined from BET measurements.

Powder Sample	BET Surface Area (m^2^/g)	Total Pore Volume (cm^2^/g)	Pore Diameter (nm)
**MgO**	72.22	0.68	33.18
**TiO_2_**	80.68	0.16	6.04

**Table 2:** Methyl orange photodegradation activity of TiO_2_ and MgO nanopowders and their band gap values.

Powder Sample	Methyl Orange UV Photodegradation (%)		Band Gap (eV)
	1 h	3 h	
MgO	56	80	4.7
TiO_2_	87	93.7	3.15

**Table 3:** Optical density at 600 nm of MgO and TiO_2_ nanopowders after 24 h.

Tested Nanopowder Concentration (0.5 mg/mL)	OD of Sample with *S. aureus*	OD of Sample without *S. aureus*	Real OD Caused by Microbial Growth
MgO	0.043	0.038	0.005
TiO_2_	0.220	0.101	0.119
Biological control of *S. aureus*	-	-	0.243

**Table 4:** Growth inhibition zone of tested microorganisms around polymer-oxide suspensions.

Polymer-Oxide Suspensions	Microorganism Used for Testing/Results in mm		
	*Staphylococcus aureus*	*Aspergillus niger*	*Candida albicans*
NaPAC_16_-MgO	11	9	7
NaPAC_16_-TiO_2_	14	6	4

**Table 5:** Area roughness of composite films deposited on glass slide substrates.

Film on Glass Slide Sample	Root Mean Square Height (µm)
Glass/5 layers NaPAC_16_ + MgO NPs (0.5%)	0.02125
Glass/5 layers NaPAC_16_ + TiO_2_ NPs (0.5%)	0.01107

**Table 6:** Static contact angle values measured on the glass slide substrate. Dip-coated glass slides (five-layer coatings)—contact angle θ (°).

Sample	Glass	Glass/5 Layers NaPAC_16_	Glass/5 Layers NaPAC_16_ + MgO	Glass/5 Layers NaPAC_16_ + TiO_2_
Contact angle θ (°)	41.26	74.50	98.79	78.50

**Table 7:** Photodegradation efficiency of MO measured on the two types of composite films.

Film on Glass Slide Sample	Methyl Orange UV Photodegradation (%)		
	1 h	3 h	5 h
Glass/1-layer NaPAC_16_	0%	0%	1%
Glass/5 layers NaPAC_16_ + TiO_2_ NPs (0.5%)	3.9%	13.62%	17.10%
Glass/5 layers NaPAC_16_ + MgO NPs (0.5%)	18.86%	36.9%	49.13%
MgO NPs	56%	80%	-
TiO_2_ NPs	87%	93.7%	-

**Table 8:** Colorimetric parameters measured on the stone fragments before and after immersion coating.

Sample	L*	a*	b*	ΔL*	Δa*	Δb*	ΔEx	ΔE
Limestone 1	83.24	1.02	1.03	−10.18	1.57	−2.28	10.54	
Limestone 1 coated with MgO-NaPAC_16_	82.86	1.19	0.99	−10.56	1.73	−2.31	10.95	0.41
Limestone 2	84.39	0.99	3.74	−9.03	1.54	0.44	9.17	
Limestone 2 coated with TiO_2_—NaPAC_16_	84.14	1.14	3.97	−9.28	1.69	0.67	9.46	0.29

**Table 9:** Colorimetric parameters measured on the stone fragments before and after immersion in coating.

Sample	L*	a*	b*	ΔL*	Δa*	Δb*	ǀΔb*ǀ	Δbx	ΔEx	ΔEx’
M1(a) reference	85.85	0.46	1.58	−7.57	1.01	−1.72	1.72		7.83	
M1(a) MgO—NaPAC_16_	86.06	0.62	1.49	−7.36	1.17	−1.81	1.81	0.09	7.67	0.16
M1(a) TiO_2_—NaPAC_16_	86.19	0.70	1.63	−7.23	1.25	−1.68	1.68	−0.03	7.53	0.30
M1(b) reference	77.85	2.12	1.13	−15.57	2.67	−2.17	2.17		15.95	
M1(b) MgO—NaPAC_16_	78.13	2.29	0.82	−15.29	2.84	−2.48	2.48	0.11	15.74	0.21
M1(b) TiO_2_—NaPAC_16_	78.57	2.28	0.92	−14.85	2.83	−2.39	2.39	0.22	15.31	0.64

**Table 10:** Colorimetric parameters measured on red bricks samples before and after 15 min immersion in specific dispersion.

Sample	L*	a*	b*	ΔL*	Δa*	Δb*	ǀΔb*ǀ	Δbx	ΔEx	ΔE
Reference specimen	76.44	4.35	1.31	−16.98	4.90	−2.00	2.00	-	17.79	-
MgO + NaPAC_16_	76.28	4.52	1.48	−17.14	5.07	−1.83	1.83	−0.18	17.97	0.18
TiO_2_ + NaPAC_16_	76.06	4.45	1.25	−17.36	5.01	−2.05	2.05	0.05	18.18	0.39

## Data Availability

Data is contained within the article.
